# Donation after brain death followed by circulatory death, a novel donation pattern, confers comparable renal allograft outcomes with donation after brain death

**DOI:** 10.1186/s12882-018-0972-8

**Published:** 2018-07-04

**Authors:** Qipeng Sun, Honglan Zhou, Ronghua Cao, Minzhuan Lin, Xuefeng Hua, Liangqing Hong, Zhengyu Huang, Ning Na, Ruiming Cai, Gang Wang, Fanhang Meng, Qiquan Sun

**Affiliations:** 1 Department of Renal Transplantation, The Third Affiliated Hospital, Sun Yat-sen University, Kaichuang Road 2693, Huangpu District, Guangzhou, 510530 People’s Republic of China; 2 Department of Urology, The First Affiliated Hospital, Jilin University, Xinmin Road 71, Changchun, 130000 People’s Republic of China; 3 Department of Renal Transplantation, The Second Affiliated Hospital, Guangzhou Traditional Chinese Medicine University, Inner Ring Road 55, University City, Guangzhou, 510280 People’s Republic of China; 4 Department of Renal Transplantation, The Third Affiliated Hospital, Guangzhou Medical University, Duobao Road 63, Guangzhou, 510530 People’s Republic of China

**Keywords:** Donation after brain death followed by circulatory death, Donation after brain death, Delayed graft function, Acute rejection

## Abstract

**Background:**

Organ donation after brain death (DBD) is the standard strategy for organ transplantation; however, the concept of brain death is not universally accepted due to cultural beliefs and barriers amongst billions of people worldwide. Hence, a novel donation pattern has been established in China which outlines the concept of donation after brain death followed by circulatory death (DBCD). Differently from any current donation classification, this new concept is formulated based on combination of recognizing brain death and circulatory death. Should approval be gained for this definition and approach, DBCD will pave a novel donation option for billions of people who cannot accept DBD due to their cultural beliefs.

**Methods:**

A multi-center, cohort study was conducted from February 2012 to December 2015. 523 kidney transplant recipients from four kidney transplant institutions were enrolled into the study, of which, 383 received kidneys from DBCD, and 140 from DBD. Graft and recipient survivals following transplantation were retrospectively analyzed. Postoperative complications including delayed graft function,, and acute rejection, were also analyzed for both groups.

**Results:**

DBCD could achieve comparable graft and recipient survivals in comparison with DBD (Log-rank *P* = 0.32 and 0.86,respectively). One-year graft and recipient survivals were equal between DBCD and DBD groups (97.4% versus 97.9%, *P* = 0.10;98.4% versus 98.6%, *P* = 1.0, respectively). Furthermore, DBCD did not increase incidences of postoperative complications compared with DBD, including delayed graft function (19.3% versus 22.1%, *P* = 0.46) and acute rejection (9.1% versus 8.6%, *P* = 1.0). Additionally, antithymocyte globulin as induction therapy and shorter warm ischemia time decreased incidence of delayed graft function in DBCD group (16.8% on antithymocyte globulin versus 27.2% on basiliximab, *P* = 0.03; 16.7% on ≤18 min versus 26.7% on > 18 min group, *P* = 0.03).

**Conclusions:**

Kidney donation through DBCD achieves equally successful outcomes as DBD, and could provide a feasible path to graft availability for billions of people who face barriers to organ donation from DBD.

## Background

Kidney transplantation is currently the preferred treatment mode for patients with end-stage renal disease [1]. Although kidney donation after brain death (DBD) is considered the standard donation strategy and has been established in Western countries, there are many obstacles preventing acceptance of the Harvard criteria for brain death in some countries, such as China and Japan [2–6]. In many of these countries, such as Japan, living kidney donations from a relative often have to be used to relieve pressure placed on organ sources, but ethical and serious constraints cause controversy in these instances [2, 7]. Although donation after circulatory death (DCD) has the potential to increase kidney transplants, inferior long-term survival of recipients from DCD, and a higher incidence of delayed graft function (DGF) make clinicians reluctant to accept DCD transplantations [8, 9]. Hence, establishing a novel donation path for these large populations unable to accept DBD is now critical.

In the light of cultural barriers, efforts to promote DBD in China have not been satisfied from 2009 [10]. Hence, a novel donation concept namely organ donation after brain death followed by circulatory death (DBCD) was officially initiated in 2011, which could be regarded as combination of recognizing brain death and circulatory death [11, 12]. These donors should first be declared as brain dead, and then processed with planned withdrawal of mechanical support, and subsequent execution of cardiac death protocols, which is totally different from the international common practice of Maastricht criteria of donation after cardiac death [11, 12]. Should approval be gained for this definition and approach, DBCD will pave a novel donation option for billions of people who cannot accept DBD due to their cultural beliefs. To this end, we conducted a multi-center cohort study to compare outcomes from kidney donation through DBCD compared with DBD.

## Methods

### Study design

This was a multi-center, retrospective, observational cohort study involving 523 kidney transplants from DBCD or DBD from February 2012 to December 2015. The transplants were performed in four institutions: The Third Affiliated Hospital of Sun Yat-sen University, The First Affiliated Hospital of Jilin University, The Second Affiliated Hospital of Guangzhou Traditional Chinese Medicine University, and The Third Affiliated Hospital of Guangzhou Medical University. Of the 523 kidney transplant recipients enrolled, 383 received DBCD and 140 DBD.

Donors were selected based on confirmed patient identity, age ≤ 65 years, no history of kidney disease, drug abuse or uncontrollable psychotic symptoms, no active infection including HIV, bacteria or fungus, no history of uncontrolled hypertension, diabetes mellitus with complications, no history of malignant melanoma, metastatic or incurable malignancy [11].

No organs from executed prisoners were used in the study, and procurement of kidneys from all donors was conducted in accordance with the Declaration of Helsinki and Istanbul Declaration, and approved by the Human Organ Transplantation and Ethics Committee of each institution. The donation types were determined by donors’ families, who also signed consent forms that were approved by the Ethics Committees of each institution. The Ethics Committees were established according to the Operational Guidelines for Ethics Committees that Review Biomedical Research developed by the World Health Organization, and consequently included physicians, lawyers, statisticians, paramedical personnel, and laypersons [13]. Organ allocation was conducted equitably and transparently by the China Organ Transplant Response System [3].

The definition of brain death was strictly determined by the exclusion of confounders, as well as the presence of three essential findings; namely, irreversible coma, absence of spontaneous motor activity, and the absence of all brain-stem reflexes. To determine the cessation of respiratory function, an apnea test should be carried out before declaration of brain death as follows: after mechanical ventilation and vasopressors withdrawn, 100% concentration of O_2_ with a velocity of 6 L/minute was added to patient via tracheal intubation for 8–10 min. Breathing movement should be closely observed and arterial blood gas analysis should be tested finally. The cessation of spontaneous respiratory function was defined as no breathing movement with PaCO_2_ equal or greater than 60 mmHg or a rise of 20 mmHg more than basal level. The brain death of each donor was adjudicated independently by two neurologists or neurosurgeons [5, 11].

Cardiac death was legally defined as irreversible cessation of circulatory function, based on definitive proof by confirmatory tests, such as electrocardiography, intra-arterial monitoring or Doppler ultrasound. An observation period of 5 min was also employed, to ensure the irreversibility and permanence of the patient’s cardiac death [11].

### Organ procurement and management

Organ donation and recovery was conducted by organ procurement organization established by the National Health and Family Planning Commission of China. DBDs were procured after declaration of brain death according to the aforementioned diagnostic criteria. For DBCDs, following determination of brain death, written consent was obtained from the donor’s immediate family, agreeing to donation of the kidney and withdrawal of life support. The obtained consent for donation was then reported to the Organ Donation Committee, which supervised the DBCD process.

**Fig. 1:**
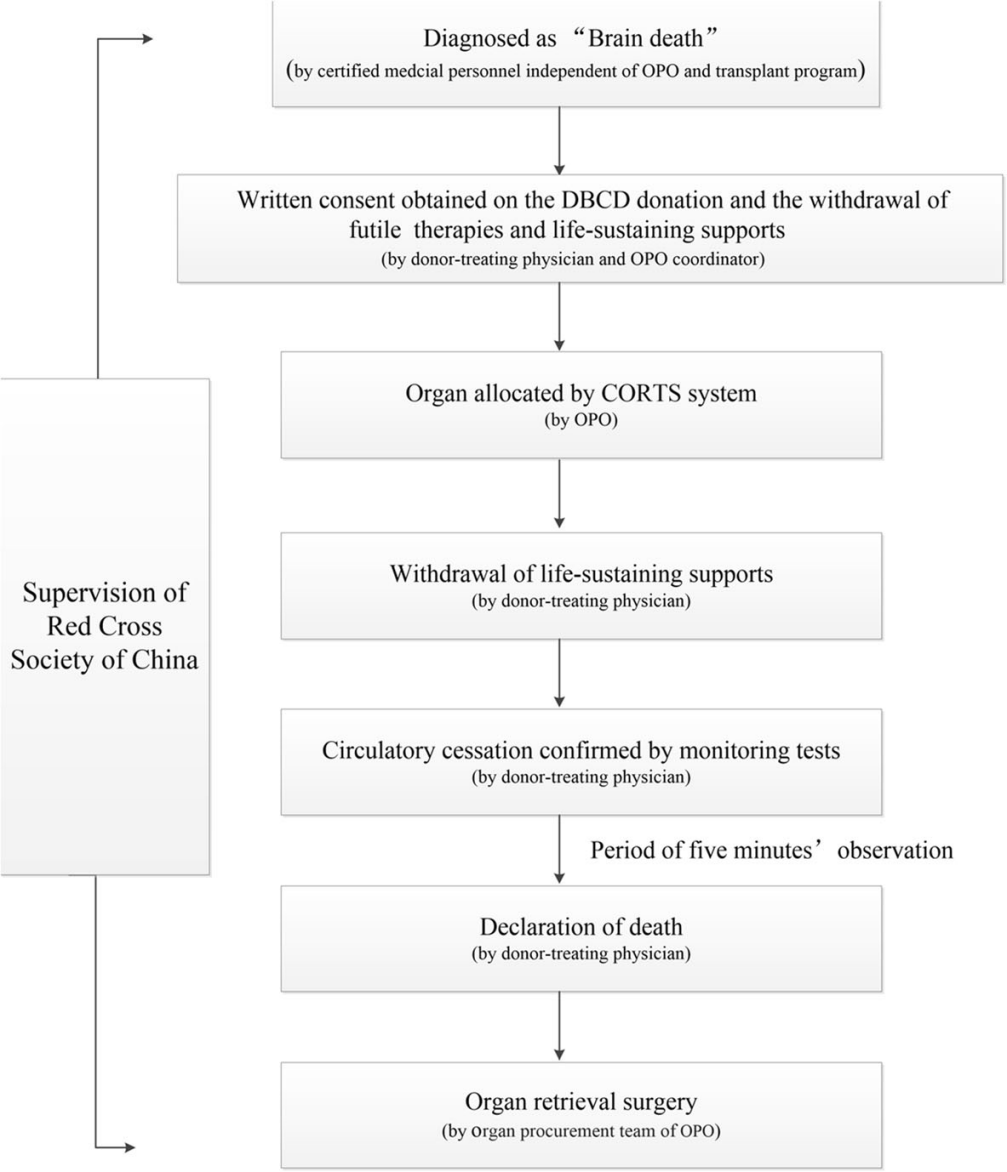
The protocol for organ DBCD in China

Donors were monitored with invasive blood pressure sensors in the operating room. Following condolences, mechanical ventilation and vasopressors were withdrawn, and vital signs were continued to be monitored. The definition of cardiac death was determined according to the aforementioned criteria, and after 5 min of observation following cardiac arrest, death was declared and organ recovery initiated. According to the national guidelines in China, warm ischemia time was recorded from the termination of life support to the hypothermic perfusion of graft, and cold ischemia time was recorded as the duration from hypothermic perfusion to blood reperfusion during transplantation surgery. The warm ischemia time of the kidney grafts was limited to under 60 min [11]. Zero-time biopsies were also performed when deemed necessary. The protocol for DBCD in China was shown in Fig. 1.

### Immunosuppressive regimen

Either antithymocyte globulin (ATG) or basiliximab was administered as induction therapy. Additionally, methylprednisolone (500 mg/day) was continuously administered intravenously during the first 3 days postoperatively. Maintenance immunosuppressive regimens consisted of a calcineurin inhibitor, mycophcnolate mofetil, and prednisone. Myeophenolate mofetil initiated immediately after transplantation was maintained at a daily dose of 1.0–1.5 g. Tacrolimus or cyclosporine was started on Day 2–4 at 0.1–0.15 mg/kg/day or 6–8 mg/kg/day, respectively, according to the level of recovery of renal graft function. The immunosuppressive regimens were adjusted to achieve target therapeutic trough levels in peripheral blood. Oral administration of prednisone, initiated at 30 mg/day on Day 4 following transplantation, was reduced by 5 mg every week to a maintenance dose of 10–15 mg/day.

### Postoperative complications

Postoperative complications such as incidences of DGF, primary non-function (PNF) and acute rejection (AR) were retrospectively analyzed in this study. The definition of DGF in our study is the use of dialysis in the first postoperative week, or failure of serum creatinine to decrease by 10% in the first 48 h following transplantation [14]. PNF was defined as failure of the transplanted kidney function, and the need for dialysis therapy [15]. Recipients with primary non-function were excluded from delayed graft function defined as need for dialysis after transplantation. AR was identified upon analysis of a biopsy and classified according to the Banff 2013 classification [16]. Human leukocyte antigen (HLA) mismatching between donor and recipient was categorized according to differences at the HLA-A, HLA-B, and HLA-DR loci;: 0–1 of six possible mismatches, 2–4 mismatches, and 5–6 mismatches [17]. Positive panel-reactive antibody (PRA) was defined as peak PRA more than 10% in our study, and a categorical variable for positive PRA was made as following: peak PRA 10–50%, 50–80%, and > 80%. In addition to the above, other severe postoperative complications including complicated urinary tract infection, severe pneumonia, severe bleeding, anastomotic stenosis of the ureter/bladder, renal allograft rupture, lymphorrhagia and urine leakage were also analyzed retrospectively.

### Follow-up after kidney transplantation from DBCD and DBD

All recipients were followed up and routine tests as well as concentration of calcineurin inhibitors were regularly assessed. Graft and recipient survivals were also analyzed. Follow-up of a patient ceased upon the patient’s death, PNF, resection of the transplanted kidney or secondary transplantation due to complications arising from the first.

### Statistical analysis

Baseline characteristics of recipients and donors as well as postoperative complications between the two groups were compared using the t-test for continuous variables or the chi-squared test for discrete variables. Kaplan–Meier curves were plotted to depict graft and recipient survivals, and a curve comparison was performed between the two groups using the log-rank test. Incomplete data entries were not included in our analyses. Risk factors for survivals were studied using a cox regression regression analysis. All analyses were performed with Statistical Package for Social Science 22.0 for Windows (IBM Corp., Armonk, NY, USA). *P* values less than 0.05 were considered statistically significant.

## Results

### Baseline characteristics of donors and recipients

In this study, 199 DBCD and 85 DBD donors were included. There was no significant difference in gender, use of vasoactive drugs, cardio-pulmonary resuscitation, intensive care unit time, body mass index and serum terminal creatinine between the two groups (all *P* > 0.05) (Table 1). No significant difference was also found in prevalence of arterial hypertension, heart disease, or diabetes (all *P* > 0.05). Although no significant difference was found in mean age between two groups, 59.8% of DBCD donors were older than 40 years, compared with 12.1% of DBD donors (*P* < 0.05). A significant difference was found in the causes of death and a significantly greater proportion of DBD donors died from cerebral trauma than DBCD donors (69.4% versus 53.3%, *P* = 0.01). Likewise, a significantly greater proportion of DBCD donors died from cerebrovascular accident than DBD donors (42.2% versus 27.1%, *P* = 0.02). The warm ischemia time of DBCD donation was 16.2 ± 5.2 min, with a range of 9 to 35 min. Among different groups of warm ischemia time, 57.8% donors had warm ischemia time 10-20 min. No significant difference was found in cold ischemia time between the two groups (5.5 ± 2.2 in DBCD versus 5.7 ± 2.4 h in DBD, *P* = 0.06).

**Table 1:** Demographics of donors included in this study

Clinical Values	DBCD (*n* = 199)	DBD (*n* = 85)	*P*
Age(years)	40.4 ± 13.0	28.8 ± 11.6	0.26
Gender (%Female)	15.1	15.3	1
Cause of death, n (%)			
Cerebral trauma	106(53.3)	59(69.4)	0.04
Cerebrovascular accident	84(42.2)	23(27.1)	
Others	9(4.5)	3(3.5)	
Using of vasoactive drugs, n (%)	153(76.9)	71(83.5)	0.27
Cardio-pulmonary resuscitation, n (%)	17(8.5)	7(8.2)	1.00
ICU Time of Donor(days)	6.8 ± 5.7	8.1 ± 6.7	0.14
Donor BMI (kg/m^2^)	22.9 ± 2.2	21.5 ± 3.6	0.11
Terminal donor Cr(μmol/L)	149.9 ± 55.9	147.4 ± 123.3	0.90
History of arterial hypertension, n (%)	43(21.6)	11(12.9)	0.10
History of heart disease, n (%)	6(3.0)	5(5.9)	0.31
History of diabetes, n (%)	6(3.0)	1(1.2)	0.68
Cold ischemia time(hours)	5.5 ± 2.2	5.7 ± 2.4	0.06
Warm ischemia time(minutes)	16.2 ± 5.2	–	–
< 10 min	39(19.6)	–	–
10–20 min	115(57.8)	–	–
20-30 min	37(18.6)	–	–
> 30 min	8(4.0)	–	–

*ICU* intensive care unit, *BMI* body mass index, *Cr* creatinine

No significant differences were found between recipients from the two groups for most baseline characteristics (*P* > 0.05) (Table 2). The majority of recipients received their first transplantations in this study. Panel-reactive antibody (PRA) was positive in 12 (3.1%) of recipients in the DBCD group, compared with five (3.6%) in the DBD group, although this was not statistically different (*P* = 0.78). All the positive PRA recipients in the two groups were classified into 10–50%. Regarding induction therapy, 291 recipients (76%) in the DBCD group received ATG compared with 39 recipients (27.9%) in the DBD group (*P* < 0.05).

**Table 2:** Demographics of recipients included in this study

Clinical Values	DBCD (*n* = 383)	DBD (*n* = 140)	*P*
Age(years)	42.5 ± 11.1	43.1 + 11.6	0.85
Gender (%Female)	37.6	32.1	0.26
Preoperative Cr(μmol/L)	931.2 ± 287.4	1033.6 ± 324	0.06
Previous transplants, n (%)			
First transplant	374(97.7)	135(96.4)	0.54
Second transplant	9(2.3)	5(3.6)	
PRA, n (%)			
< 10%	371(96.9)	135(96.4)	0.78
10–50%	12(3.1)	5(3.6)	
Induction therapy, n (%)			
antithymocyte globulin	291(76)	39(27.9)	0
basiliximab	92(24)	101(72.1)	
HLA mismatches, n (%)			
0–1	274(71.5)	106(75.7)	0.22
2–4	79(20.6)	29(20.7)	
5–6	30 (7.9)	5(3.6)	

*Cr* creatinine, *PRA* panel-reactive antibody, *HLA* human leukocyte antigen

### Postoperative complications

There was no significant difference in postoperative complications between the two groups (all *P* > 0.05; Table 3). Of the DBCD recipients, 19.3% developed DGF 1 week postoperatively, compared with 22.1% of DBD recipients (*P* = 0.46). Five recipients in DBCD group developed PNF, but none from DBD group (*P* = 0.33). However, all the five grafts with PNF were confirmed with severe glomerulosclerosis by zero-time biopsy. ATG was found to be related with lower incidence of DGF in comparison to basiliximab in the DBCD group (16.8% with DGF on ATG versus 27.2% with DGF on basiliximab, *P* = 0.03). Additionally, 9.1% cases from DBCD group suffered from AR compared with 8.6% in DBD group (*P* = 1.0). According to a previous published study, we divided warm ischemia time into two groups: ≤18 min and > 18 min [18]. Compared with the ≤18 min group, 54 donors (23.7%) had WIT> 18 min, of whom 17(31.5%) had DGF. (31.5% versus 15.2%, *P* = 0.02). Of 383 recipients, 101 patients (26.4%) received grafts with WIT > 18 min, of whom there were 27 patients (26.7%) suffered from DGF (26.7% versus 16.7%, *P* = 0.03). Incidences of PNF and AR were also higher in the > 18 min group; however, no significant difference was found (3.1% versus 0.7% of PNF, *P* = 0.12; 13.5% versus 8.9% of AR, *P* = 0.31). Serum creatinine levels were recorded at different follow-up time points, and no significant difference was found in serum creatinine curves between the two groups (*P* = 0.08).

**Table 3:** Postoperative complications between DBCD and DBD

Clinical Values	DBCD (*n* = 383)	DBD (*n* = 140)	*P*
DGF, n (%)	74(19.3)	31(22.1)	0.46
PNF, n (%)	5(1.3)	0(0)	0.33
AR, n (%)	35(9.1)	12(8.6)	1.00
Complicated urinary tract infection, n (%)	32(8.4)	10(7.1)	0.72
Severe pneumonia, n (%)	45(11.7)	15(10.7)	0.88
Severe bleeding, n (%)	11(2.9)	5(3.6)	0.77
Anastomotic stenosis of the ureter-bladder, n (%)	3(0.8)	2(1.5)	0.61
Renal allograft rupture, n (%)	3(0.8)	2(1.5)	0.614
Lymphorrhagia or urine leakage, n (%)	12(3.2)	7(5)	0.30
Other, n (%)	33(8.8)	8(5.7)	0.36

*DGF* delayed graft function, *PNF* primary non-finction, *AR* acute rejection

### Graft and patient outcomes following kidney transplantation from DBCD and DBD

**Fig. 2:**
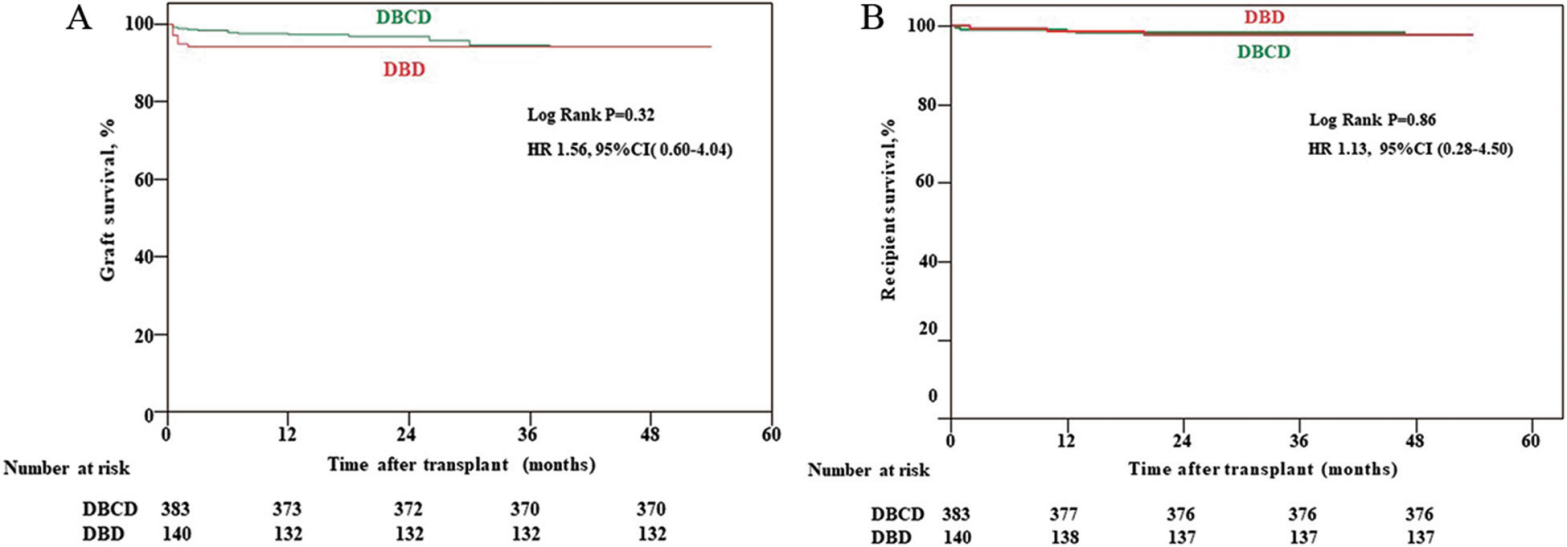
Kaplan-Meier survival estimates after renal transplantation in recipients of kidneys from DBCD and DBD. **a** Graft survival in the DBCD group was comparable to the DBD group (*P* = 0.32), HR1.56, 95% CI 0.60–4.04. **b** A similar analysis revealed no significant difference in recipient survival between DBCD and DBD groups (*P* = 0.86), HR1.13, 95% CI 0.28–4.50)

The median follow-up time for graft and recipient was 25 months (range 0.5–54 months, and 2–54 months, respectively). Graft survival in DBCD group was comparable to DBD group (*P* = 0.32; Fig. 2a). A similar analysis revealed no significant difference in recipient survival between the two groups (*P* = 0.86; Fig. 2b). One-year graft and recipient survivals were comparable between DBCD and DBD groups (97.4% versus 97.9%, *P* = 0.10 and 98.4% versus 98.6%, *P* = 1.0, respectively). In DBCD group, five patients had PNF, five severe bleeding and three renal allograft ruptures postoperatively and resected transplanted kidneys, whereas four patients had severe bleeding, two renal allograft rupture, one peri-renal abscess, and one severe AR from DBD. Regarding recipient survival, seven from DBCD group died from severe pneumonia or cardiovascular disease, and three from the DBD group died from severe pneumonia or myocardial infarction.

The cox regression regression analysis showed that no characteristics of recipients and donors in this study affected graft and recipient survivals following kidney transplantation from DBCD and DBD (*P* > 0.05).

## Discussion

While brain death is recognized by the law in most Western countries, this concept is not readily accepted in Chinese society [19]. However, Chinese culture does recognize circulatory death, which formed the premise for the initiation of DCD [11]. The DCD guidelines were developed by the Chinese Society of Organ Transplantation to make recommendations that are ethically, legally, and medically acceptable in the practice of DCD [11]. However, a difference with the international common practice of deceased organ donation is the presence of an additional category III DBCD in the Chinese classification [10]. This new concept of donation is formulated based on the combination of the international practice of recognizing brain death and circulatory death, which is different from the international common practice of Maastricht criteria of donation after cardiac death [11]. Inclusion of DBCD aims to boost the number of eligible organ donations while ensuring minimal ischemic injury of organs [10].

To our knowledge, this is the first multi-center cohort study to assess the outcomes of DBCD compared with DBD, showing that kidney transplantation from DBCD method confers comparable results with DBD. These findings suggest that DBCD is a viable graft accessibility option; thus may pave the way for a novel donation path for billions of people that face barriers to DBD.

In our study, graft and recipient survivals, as well as incidences of postoperative complications were equally comparable among DBCD and DBD. The 1-year graft survival of 97.4% in DBCD group was higher than previously reported values of 87% in DBD, and 85% in DCD [8, 9]. Previous studies reported recipient survival of 95% in DBD and DCD kidney transplantation at 1 year postoperatively, inferior to 98.4% in our DBCD group [20]. Incidence of DGF following DBD has been reported as around 24.9%, and DCD grafts have twice the risk of developing DGF, all of which were superior to 19.3% in DBCD [8, 9, 21, 22]. An increase in serum creatinine following transplantation is indicative of the presence of acute kidney injury (AKI) and DGF. Therefore, renal function recovery postoperatively can be understood through serum creatinine change curves. We compared serum creatinine curves postoperatively, and no significant difference was found, indicating similar renal function recovery between DBCD and DBD. Incidence of PNF in the DBCD group was equivalent to DBD in our study, which was also lower than reported value of 3% in DBD and DCD [9]. Previous studies have attributed higher incidence of PNF to the irreversible warm ischemic injury and perivascular edema of capillaries in DCD processes, which can result in high risk of PNF [21, 22]. However, the five grafts with PNF in DBCD group, only resulted because of severe glomerulosclerosis, confirmed through zero-time graft biopsy, and not through warm ischemia injury during organ procurement of DBCD. As for AR, there was a similar result among the two groups, which was also inferior to reported incidences of 24% in DBD and 16% in DCD [9].

The fundamental cause of comparable outcomes lies in controlled warm ischemia time during procurement of DBCD grafts. With regards to DCD, kidney is susceptible to prolonged warm ischemic injuries and severe anaerobic metabolism which results in increased risk of chronic allograft nephropathy and acute tubular necrosis. Prolonged warm ischemia time also causes further severe events related to ischemia and reperfusion injury (IRI) as well as AKI, all of which inevitably exert adverse effects on graft and recipient survival, and increase incidences of DGF and PNF [9, 22, 23]. However, DBCD in the case of planned protocols to withdraw mechanical support following brain death, can effectively limit warm ischemia time to an appropriate range. In our study, the average warm ischemia time of kidney from DBCD was limited to 16.2 ± 5.2 min, much lower than an average of 27 min through DCD [24]. Furthermore, we found higher incidences of DGF, PNF and AR in warm ischemia time of more than 18 min. We suppose that a controlled warm ischemia period of the DBCD process contributes to reducing IRI, and leads to better graft function recovery and survival. The controlled ischemia time also reduces production of chemokines or cytokines, and T cells into the graft, all of which can reduce AR [25, 26]. Additionally, lower incidences of DGF and AR in DBCD exert a positive effect on graft and recipient survival.

A standard protocol defines the approval criteria for DBCD. Before declaration of brain death, an apnea test should be carried out to determine whether autonomous respiration exits. The test should be terminated if cardiac arrhythmia, decline of oxygen saturation and blood pressure are observed. In order to limit warm ischemic time, cardio-respiratory support should be withdrawn in the operating room after all necessary preparations have been completed, and the retrieval team and operating room staff should be ready in advance [11]. ATG is recommended as induction therapy in DBCD organ transplantation; in our study, ATG was used more frequently in recipients of DBCD donations and related with a lower incidence of DGF in comparison to basiliximab. A previous study has reported 1-year graft survival of 96.9% for ATG, and 75.9% for basiliximab [27]. Other studies also demonstrated improved trend in patient and graft survivals, as well as significantly lower rates of DGF, AR, and postoperative infections in ATG-induced DCD recipients [28–30]. Thus, these findings support the positive effect of ATG in decreasing DGF and AR in our study.

Through comparison of donor baseline characteristics, 59.8% of DBCD donors were identified to be older than 40 years, compared with 12.1% of DBD donors (*P* < 0.05), this may be attributed to the more conservative view held by older people in China regarding brain death, and the understanding of younger generation in DBD. Interestingly, a significantly higher proportion of DBCD donors died from cerebrovascular episodes. Age of a donor may have influenced this, as older people are potentially at a higher risk of developing cerebrovascular events.

Information about the long-term outcome of DBCD was not available in our study due to the limited time since which this novel donation pattern had been established. Additionally, more accurate measurement of warm ischemia time of DBCD should be ensured, which could account for the unexpected finding that PNF and AR were higher in the > 18 min group, although no significant difference was found. Warm ischemia time was reported no effect on graft outcome which may be attributed to insufficient samples size and inability to detect this effect [9, 31]. Thus, future, larger prospective studies are necessary to validate our findings, and standardized protocols for donor monitoring and data collection will improve the quality and completeness of data.

DBCD, a novel donation approach, provides an acceptable outcome in terms of both survival and postoperative complications, and should be regarded as equivalent to DBD. The benefits of DBCD largely arise from its controlled procurement process of grafts which reduces the risks of warm ischemia injury. The controlled processes of DBCD should therefore be strictly followed.

## Conclusions

In conclusion, although DBD is currently the standard strategy for organ transplantation, our findings suggest that DBCD is a viable option to provide graft availability to renal transplant patients. In view of the large numbers of people universally who cannot accept DBD, DBCD can be considered a cultural and medical milestone that overcomes donation barriers.

## Abbreviations

AKI: Acute kidney injury
AR: Acute rejection
ATG: Antithymocyte globulin
DBCD: Donation after brain death followed by circulatory death
DBD: Donation after brain death
DCD: Donation after circulatory death
DGF: Delayed graft function
HLA: Human leukocyte antigen
IRI: Ischemia and reperfusion injury
PNF: Primary non-function
PRA: Panel-reactive antibody
